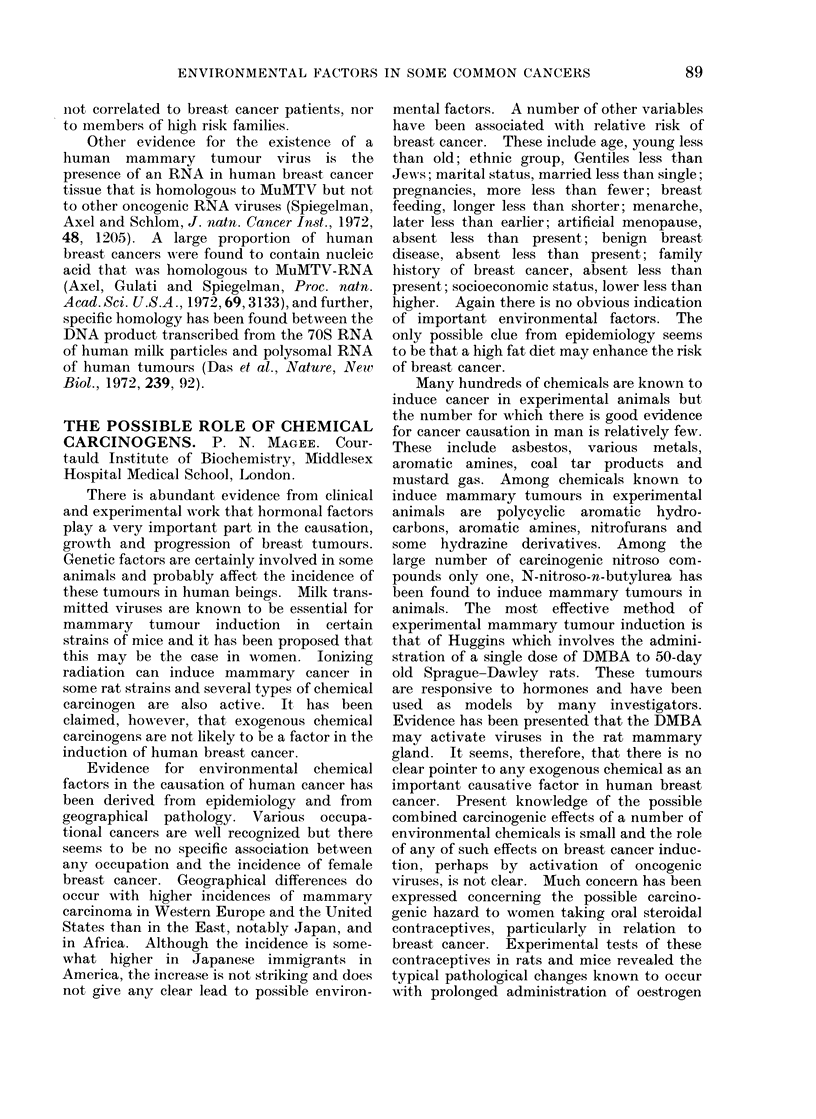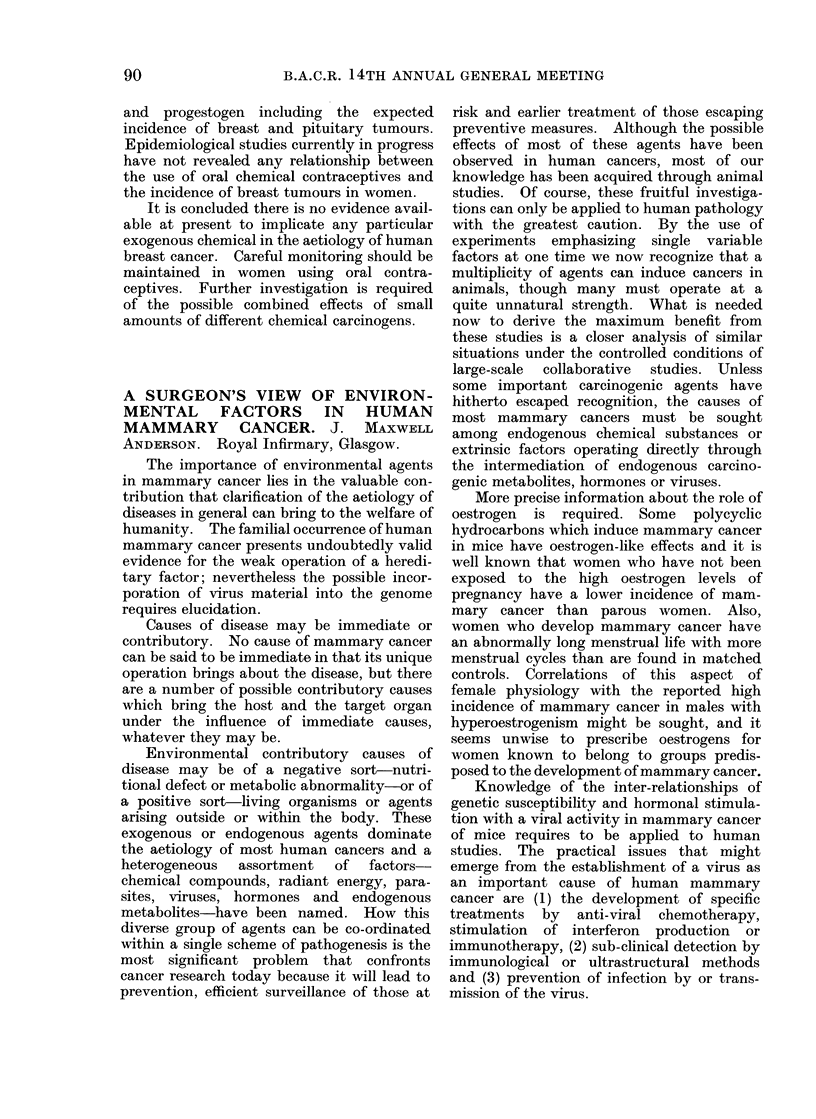# The possible role of chemical carcinogens.

**DOI:** 10.1038/bjc.1973.116

**Published:** 1973-07

**Authors:** P. N. Magee


**THE POSSIBLE ROLE OF CHEMICAL CARCINOGENS.** P. N. MAGEE. Courtauld Institute of Biochemistry, Middlesex Hospital Medical School, London.

There is abundant evidence from clinical and experimental work that hormonal factors play a very important part in the causation, growth and progression of breast tumours. Genetic factors are certainly involved in some animals and probably affect the incidence of these tumours in human beings. Milk transmitted viruses are known to be essential for mammary tumour induction in certain strains of mice and it has been proposed that this may be the case in women. Ionizing radiation can induce mammary cancer in some rat strains and several types of chemical carcinogen are also active. It has been claimed, however, that exogenous chemical carcinogens are not likely to be a factor in the induction of human breast cancer.

Evidence for environmental chemical factors in the causation of human cancer has been derived from epidemiology and from geographical pathology. Various occupational cancers are well recognized but there seems to be no specific association between any occupation and the incidence of female breast cancer. Geographical differences do occur with higher incidences of mammary carcinoma in Western Europe and the United States than in the East, notably Japan, and in Africa. Although the incidence is somewhat higher in Japanese immigrants in America, the increase is not striking and does not give any clear lead to possible environmental factors. A number of other variables have been associated with relative risk of breast cancer. These include age, young less than old; ethnic group, Gentiles less than Jews; marital status, married less than single; pregnancies, more less than fewer; breast feeding, longer less than shorter; menarche, later less than earlier; artificial menopause, absent less than present; benign breast disease, absent less than present; family history of breast cancer, absent less than present; socioeconomic status, lower less than higher. Again there is no obvious indication of important environmental factors. The only possible clue from epidemiology seems to be that a high fat diet may enhance the risk of breast cancer.

Many hundreds of chemicals are known to induce cancer in experimental animals but the number for which there is good evidence for cancer causation in man is relatively few. These include asbestos, various metals, aromatic amines, coal tar products and mustard gas. Among chemicals known to induce mammary tumours in experimental animals are polycyclic aromatic hydrocarbons, aromatic amines, nitrofurans and some hydrazine derivatives. Among the large number of carcinogenic nitroso compounds only one, N-nitroso-*n*-butylurea has been found to induce mammary tumours in animals. The most effective method of experimental mammary tumour induction is that of Huggins which involves the administration of a single dose of DMBA to 50-day old Sprague–Dawley rats. These tumours are responsive to hormones and have been used as models by many investigators. Evidence has been presented that the DMBA may activate viruses in the rat mammary gland. It seems, therefore, that there is no clear pointer to any exogenous chemical as an important causative factor in human breast cancer. Present knowledge of the possible combined carcinogenic effects of a number of environmental chemicals is small and the role of any of such effects on breast cancer induction, perhaps by activation of oncogenic viruses, is not clear. Much concern has been expressed concerning the possible carcinogenic hazard to women taking oral steroidal contraceptives, particularly in relation to breast cancer. Experimental tests of these contraceptives in rats and mice revealed the typical pathological changes known to occur with prolonged administration of oestrogen and progestogen including the expected incidence of breast and pituitary tumours. Epidemiological studies currently in progress have not revealed any relationship between the use of oral chemical contraceptives and the incidence of breast tumours in women.

It is concluded there is no evidence available at present to implicate any particular exogenous chemical in the aetiology of human breast cancer. Careful monitoring should be maintained in women using oral contraceptives. Further investigation is required of the possible combined effects of small amounts of different chemical carcinogens.